# *In Vitro* Evaluation of Intestinal Barrier Function after Exposure to Digested Pea Ingredients—Food Matrix Effect

**DOI:** 10.1021/acs.jafc.4c09963

**Published:** 2024-12-16

**Authors:** Marie-Hélène Perruchot, Gaëlle Boudry, Frédérique Mayeur-Nickel, Maurane Grondin, Sandra Wiart-Letort, Linda Giblin, Myriam M.-L. Grundy

**Affiliations:** † PEGASE, INRAE, Institut Agro, Saint Gilles 35590, France; ‡ NUMECAN, INSERM, INRAE, Université de Rennes, Saint Gilles 35590, France; § Teagasc Food Research Centre, Moorepark, Fermoy, Co Cork P61 C996, Ireland

**Keywords:** *Pisum sativum* L., dietary fiber, in vitro gastrointestinal digestion, gut barrier function, IPEC-J2, paracellular transport, food matrix

## Abstract

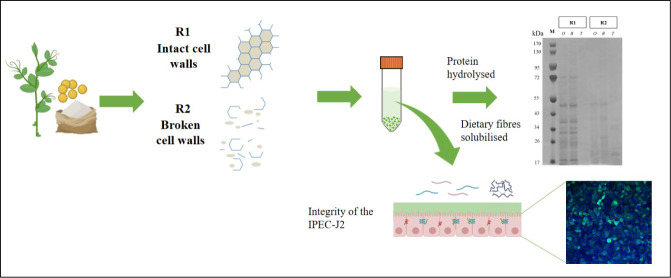

Dietary fibers (DF) are important components of human and animal diets. However, they can decrease protein digestibility and absorption and thus the nutritional value of a food. The aim of this study was to investigate how the form of delivery of pea DF impacted the integrity of the intestinal barrier and, thereby, the potential absorption of molecules. To this end, two pea flours, with either intact or ruptured cell walls, and two controls, pea fibers and pea protein, were digested *in vitro* and the digesta obtained applied onto a jejunum porcine cell line (IPEC-J2 cells). Cell viability and integrity were evaluated by transepithelial electrical resistance measurement, colorimetric assay (MTS), and immunohistochemistry for tight junction proteins. Additionally, the diffusion of FITC-dextran (FD4) and lucifer yellow (LY) through the epithelial cell monolayers was monitored. The digested pea samples did not alter the IPEC-J2 viability and permeability. For instance, no difference in the diffusion of molecules either FD4 or LY across the monolayers was observed between the different digesta and the control. Similarly, no effect was observed in ZO-1 labeling intensity compared to the control. This study demonstrated that intestinal integrity was maintained whether pea cell walls were intact or ruptured.

## 1 Introduction

Pea is a source of nutrients including proteins and dietary fibers (DF).^1^ DF can hinder protein hydrolysis and the absorption of the proteolysis products, via a range of mechanisms such as encapsulation of the protein or increase of the viscosity of intestinal content, thereby altering digestion kinetics and postprandial response.^2^ On the other hand, consumption of DF can positively influence intestinal health by providing a substrate for gastrointestinal bacteria in the colon and to a lesser extent the small intestine. Indeed, many studies have focused on the effect of DF on colonic health, in human or animals, via shaping the microbiota and the production of short chain fatty acids.^3−5^ However, DF may also promote the development of a “strong” intestinal barrier (thick mucus layer containing diverse bacterial community; cells tightly joint together). Based on DF composition and structure, DF can have different impacts on nutrients digestibility and overall intestinal health.^2,6^ One of the important parameters that influences the health impact of DF is the level of destructuration of the food matrix particularly the integrity of the plant cell wall.^6^

Digestibility studies performed in our lab with pea enabled us to estimate the level of bioaccessibility and hydrolysis of proteins as a function of plant cell wall integrity.^7,8^ Pea DF are mainly insoluble (about 70% of total DF) and composed principally of hemicellulose and pectin.^7,9^ Contrary to other seeds, such as cereals, pea DF tend not to solubilize in the gastrointestinal tract or swell when in the presence of water.^10^ Intact pea cell wall thus remain mainly undisturbed until reaching the distal part of the digestive tract.^7^ DF encapsulating proteins (intact cell walls) reduced the quantity of amino acids and small peptides potentially available for absorption. DF are, by definition, resistant to endogenous digestive enzymes present in the upper gut of humans, and more generally of monogastrics.^11,12^ Therefore, unless the DF are solubilized in the lumen of the digestive tract, and/or the plant cell walls are porous (pores large enough to let digestive enzymes penetrate inside the cell), nutrients within the plant cell are not accessible to the enzymes for hydrolysis. Questions remain regarding the fate of the products of proteolysis and how DF, under different forms, influences the integrity of the intestinal barrier and the absorption of these products. A lot of attention has been put on the effect of DF on colonic health, in human or animals, via notably the shaping of the microbiota and the production of short chain fatty acids.^3−5^ Therefore, a diet low in DF has been found to alter the gut microbiota and thereby the mucus layer that protects the intestinal mucosa in the colon.^3,13^ The functionality of the intestinal epithelium (absorption of nutrients, immunity, secretion of substances particularly mucins and enzymes) and the occurrence, or not, of diseases, relies on the integrity of the intestinal barrier.^14,15^ The direct effect of DF on this barrier in the upper gut, the primary site of absorption of proteolysis products, is not clear.

On the one hand, some studies have suggested that DF could reduce paracellular permeability possibly via the modulation of the tight junctions.^16−18^ On the other hand, DF consumption could influence intestinal morphology such as villi height, crypts depth and modulation of mucus layer.^19^ In those studies, the DF used corresponded to polysaccharides of different types, with different levels of organization, from extracted molecules to ingredients, whole foods, or even diets. However, the physicochemical properties and structure (isolated and/or solubilized polysaccharides vs intact cell wall) of the DF that transit through the gut may impact differently on intestinal epithelium integrity. Therefore, it is challenging to draw clear conclusions about the potential benefits of DF for intestinal health from these studies.

Intestinal porcine epithelial cell lines derived from the jejunum of neonatal, unsuckled piglet (IPEC-J2) can be used as an absorption model.^20,21^ Cell cultures are often used to assess the absorption rate of pharmaceutical products, micronutrients (vitamins, minerals and phenolic compounds), bacteria and virus, or toxins, especially in the colon.^22^ More recent studies have also investigated the absorption of the hydrolysis products of macronutrients (proteins, starch or lipids).^23−25^ But to the best of our knowledge, no study investigated the impact of plant cell walls, i.e., DF, on the barrier function in the upper gut where most of the absorption of hydrolyzed products takes place.

Therefore, the purpose of this work was to investigate the impact of the different pea constituents (e.g.; DF and the products of proteolysis) on intestinal epithelial cells and how the form of those compounds, especially DF, influenced IPEC-J2 viability and permeability. To do so, two pea flours, with either intact (R1) or ruptured (R2) cell walls, were digested *in vitro*. The digest was then applied onto IPEC-J2. Epithelial cell viability and integrity in the presence of the different digesta were analyzed by transepithelial electrical resistance (TEER) measurement, colorimetric assay (MTS tetrazolium reagent), and immunohistochemistry for tight junction proteins (Zonula occludens-1, ZO-1). Additionally, the diffusion of fluorescein isothiocyanate-dextran 4 kDa (FD4) and lucifer yellow (LY) across the epithelial cell monolayers was monitored. We hypothesized that a destructured pea cell wall can affect the integrity of the IPEC-J2 monolayers, and the latter will, as a consequence, become more permeable to molecules.

## 2 Materials and Methods

### 2.1 Materials and Chemicals

Pea (*Pisum sativum* L.) seeds, fibers, protein, and starch were provided by Roquette (Vic-sur-Aisne, France). The pea seeds were ground using a hammer mill fitted with a 2.5 mm grid to produce pea flour. Two flours of identical composition, but different cell wall integrity, were thus obtained (R1, ground pea seeds where proteins are encapsulated by intact cell walls) and a reconstituted pea flour (R2, “free” protein and broken cell walls; made from the combination of the extracted pea protein, fibers and starch provided by Roquette). Both flours contained 17.0% proteins and 18.2% DF (total dietary fibers); the nutritional composition of R1, R2, pea fibers (Fib; 52.1% DF and 36.1% starch) and pea protein (Prot; 71.5% proteins) have been described previously.^7^

Pepsin from porcine gastric mucosa (2145 U/mg of solid), bovine bile extract, pancreatin from porcine pancreas (3.2 U/mg of solid based on trypsin activity), and fluorescein isothiocyanate–dextran of 4 kDa were purchased from Merck (Saint Quentin Fallavier, France). The porcine jejunal intestinal cell line IPEC-J2 was obtained from DSMZ (ACC-701, Braunschweig, Germany; no information was provided by the supplier regarding passage). The cell viability assay, MTS 3-(4,5-dimethylthiazol-2-yl)-5-(3-carboxymethoxyphenyl)-2-(4-sulfophenyl)-2H-tetrazolium, inner salt reagent, was purchased from Promega. (Charbonnières-les-Bains, France). Lucifer yellow, ZO-1 monoclonal antibody (mouse) Alexa Fluor 594, wheat germ agglutinin, MUC2 polyclonal antibody, and antirabbit secondary antibody (donkey) Alexa Fluor 568 were obtained from Thermo Fisher Scientific (Illkirch-Graffenstaden, France). All other chemicals, solvents, and reagents including the media for the cell culture were from Merck (Saint Quentin Fallavier, France), unless stated otherwise.

### 2.2 *In Vitro* Digestion of Pea Ingredients

The two pea flours, R1 and R2, were digested *in vitro* as previously described,^7^ using the INFOGEST protocol which included enzyme activities measured in-house.^26^ Extracted pea fibers (Fib) and pea protein (Prot) were also used as controls to evaluate the effect of those ingredients alone on the IPEC-J2 monolayers. The amount of sample used was adjusted based on either protein (70 mg on a dry weight basis for pea protein alone, R1 or R2) or fiber (100 mg on a dry weight basis for pea fibers) content with a total volume of 8 mL at the end of the intestinal phase. At the end of the intestinal phase, enzymatic reactions were stopped by increasing the pH to 9 with 5 N NaOH. Three sets of experiments for each ingredient were carried out: incubation in simulated fluids without bile or enzymes (O), incubation with bile and without enzymes (B), and incubation with both bile and enzymes (T). Each digestion was performed in triplicate.

A detoxification step is required prior to cell experiments as proteases and bile salts will compromise cell viability.^23^ Therefore, the digesta samples (T) were diluted (1:5 and 1:10), and either heat treated at 100 °C for 5 min (1, 2, and 4 h of incubation with the digesta), or 1 mM of Pefabloc (as recommended to stop proteases activity),^23,26^ was added (only 1 h of incubation with the digesta). The samples were then centrifuged at 4,000 g for 15 min at 4 °C and the supernatant collected. The final conditions selected for detoxifying the digesta was determined as described in Section 2.7: heat treatment at 100 °C for 5 min and 1:10 dilution in media.

Bioaccessible (B samples) and hydrolyzed (T samples) proteins, expressed in %, were determined as follows:
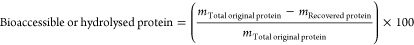
1where *m*_Recovered protein_ is the mass in g of protein recovered after *in vitro* incubation and *m*_Total original protein_ is the mass in g of protein originally present in the ingredient.

### 2.3 Estimation of Proteolysis Extent

The extent of protein hydrolysis was estimated using the OPA method with quantification of amino groups as described in Sousa et al.^27^ Briefly, the proteins and large peptides were precipitated in 80% methanol at −20 °C during 1 h, the samples centrifuged (4,000 g for 15 min at 4 °C), the supernatant was collected (50 μL) and the methanol evaporated before hydrolyzing the small peptides with hydrochloric acid (mixture of 260 μL of H_2_O, 120 μL of 0.2 M DDP (3,3′-dithiodipropionic acid) in 0.1% NaOH, 120 μL of 0.2 M HCl, and 500 μL of 37% HCl). Glutamic acid was used for the standard curve with concentrations ranging from 0 to 10 mM.

### 2.4 Dietary Fibers Observations in the Digesta

The supernatants obtained following the digestion with bile and enzymes (samples T, detoxified by heat treatment at 100 °C for 5 min and 1:10 dilution in media) were visualized using an apotome microscope and Zen software (Apotome, Zeiss, France). The fluorescent dye, Calcofluor White, was used to identify DF (solubilized cell wall fragments; 100 μL of Calcofluor White stain added to 100 μL of supernatant, 25 μL placed on a microscopy slide). Images were captured with a 10X objective lens. Samples were excited at 405 nm, and the fluorescence emitted detected at 406–460 nm.

### 2.5 Electrophoretic Profile

Proteins and peptides contained in the supernatant of the incubated O, B and T samples were separated by sodium dodecyl sulfate-polyacrylamide gel electrophoresis (SDS-PAGE) using NuPAGE Bis-Tris gels (4–12% Bis-Tris). The T, bile, and enzyme samples were detoxified by heat treatment (100 °C for 5 min) and 1:10 dilution. The enzymes sample (Enz) was also analyzed without heat treatment. For each sample, 10 μL of reducing agent (dithiothreitol, DTT; stock concentration of 77.5 mg/mL in H_2_O) and 25 μL of NUPAGE LDS sample buffer (4x) were added to 65 μL of supernatant. Then, the samples (15 μL) were loaded into the gel, and after migration (70 V for 30 min and 100 V for 1 h), the gel was stained with Coomassie Blue. PageRuler Prestained Protein was used as a molecular weight marker.

### 2.6 Cell Culture and Transepithelial Electrical Resistance (TEER) Measurement

The IPEC-J2 were grown to 80% confluence in 75 cm^2^ flasks in Dulbecco’s Modified Eagle Medium/Ham’s F-12 (DMEM/Ham’s F-12) supplemented with 10% porcine serum (PS) containing 1% penicillin-streptomycin. Then, cells at passage 4 to 7 were cultured in 5% PS containing 1% penicillin-streptomycin, 1% Insulin Transferrin Selenium (ITG), and 10 μg/mL Epithelial Growth Factor (EGF) as previously described.^21^ Cells were seeded on transwell polyester membrane inserts (0.4 μm pore size, 1.1 cm^2^ surface area), in 12-well plates at a density of 1 × 10^5^ cells per cm^2^. The cells were left to grow for 14 days in a humidified (95%) incubator at 37 °C under 5% CO_2_. The media was replaced every two to 3 days (1.5 mL in the basal compartment and 0.5 mL in the apical compartment), and the transepithelial electrical resistance (TEER) was measured on days 4, 7, 11, and 14 with an Epithelial Voltohmmeter (EVOM3, Friedberg, Germany).

TEER values, in Ω × cm^2^, were calculated as follows:
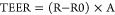
2where R is the measured resistance (Ω), R0 is the blank resistance (insert without cells), and A is the inset effective surface (1.1 cm^2^).

### 2.7 Assessment of Cell Viability (MTS Assay)

The IPEC-J2 cells were seeded (200 μL of 1 × 10^5^ cells/mL) into a 96-well microplate and left to incubate in medium (5% PS + ITG + EGF) at 37 °C under 5% CO_2_. After 24 h of incubation at 37 °C, the medium was removed and the cells washed with Hank’s Balanced Salts Solution (HBSS) before applying 100 μL of O, B and T digesta of Fib, Prot, R1 and R2, heat treated at 100 °C for 5 min plus diluted 1:5 or 1:10. Control wells included IPEC-J2 cells with HBSS, IPEC-J2 cells with simulated fluids with bile (heat treated and diluted), and IPEC-J2 cells with simulated fluids with both bile and enzymes (heat treated and diluted). The controls and digesta were removed after 1, 2, or 4 h of incubation, and the cells were washed with HBSS. Similar experiments were performed with Pefabloc but for only 1 h of incubation, as IPEC-J2 viability was affected after 1 h. After addition of 100 μL of MTS (dilution of 1:12 of the stock solution) in each well, the cells were further incubated at 37 °C for 1 h. The absorbance was then recorded at 490 nm with a microplate reader (Varioskan Lux, ThermoFisher, Villebon-sur-Yvette, France). The cell viability was calculated from the ratio of the absorbance of the cells incubated with digesta to the absorbance of the control cells (cells incubated with HBSS only).

### 2.8 Evaluation of IPEC-J2 Permeability

On day 14, the TEER was measured (T0), and the IPEC-J2 monolayers were washed with HBSS, and left to incubate for 30 min before measuring the TEER again (30 min HBSS). The HBSS was then replaced with heat-treated T digesta (0.5 mL diluted 1:10 in HBSS) in the apical compartment and with “fresh” HBSS in the basal compartment (1.5 mL). The TEER was measured immediately (0 h digesta) and after 2 h of incubation (2 h digesta).

FD4 (4 kDa) diffusion assay was carried out to evaluate the paracellular transport at day 14. FD4 solution (100 μL at 10 mg/mL in HBSS) was added to the apical compartment (total volume of 1 mL, with or without heat-treated T digesta). Media (100 μL) was removed in the basal compartment (total volume of 2 mL), and replaced with HBSS, after 15, 30, 60, and 120 min of incubation at 37 °C. Fluorescence intensity was measured using a microplate reader (Varioskan Lux, ThermoFisher, Villebon-sur-Yvette, France) at an excitation wavelength of 495 nm and an emission wavelength of 525 nm. The concentration of FD4 (ng/μL) was determined from a standard curve. These experiments were performed in triplicate. The apparent permeability coefficient of FD4 (*P*_app_, cm.s^–1^) was calculated as follows:
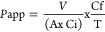
3where *V* is the volume in the basal compartment in mL, A is the surface area of the inset, Ci the initial concentration of FD4 in the apical compartment, Cf the concentration of FD4 in the basal compartment, and T the time in second.

Complementary to the FD4 assay, the passage of lucifer yellow (LY, 521.8 Da) across the epithelial cell monolayers was monitored. The culture medium was removed at day 14, and the monolayers were washed with HBSS. LY (final concentration of 200 μM) was added to the apical compartment containing HBSS or the heat-treated digesta (FibT, ProtT, R1T, and R2T). The monolayers were left to incubate at 37 °C, and 100 μL of the basal media was removed at 15, 30, 60, and 120 min and replaced with “fresh” HBSS. The fluorescence was measured at an excitation wavelength of 428 nm and an emission of 540 nm with a microplate reader (Varioskan Lux, ThermoFisher, Villebon-sur-Yvette, France). The LY concentration, in μM, was determined from a standard curve (0 to 200 μM). Each assay was repeated three time. The *P*_app_ of LY was calculated according to the eq 3.

### 2.9 Immunochemistry

After 14 days of differentiation, cell media or heat-treated T digesta diluted in 1:10 in media (Fib, Prot, R1 and R2) were added to the apical chamber of the IPEC-J2 monolayers. After 2 h of incubation, the apical and basolateral compartment were emptied, and the monolayers were fixed with methanol, washed twice in phosphate-buffered saline (PBS) solution, permeabilized with Triton X100 and washed twice again with PBS. The monolayers were then incubated for 1 h 30 at 37 °C with a primary anti-ZO-1 antibody conjugated with Alexa Fluor 594 (100 μL), diluted 1:100 in PBS-0.2% BSA. The samples were mounted on microscopy slides with 15 μL of ProLong Gold Antifade Mountant with DNA Stain 4′,6-diamidino-2-phenylindole (DAPI) to stain the nuclei.

For the mucin production, after fixation and permeabilization, the monolayers were incubated with either wheat germ agglutinin or MUC2 polyclonal antibody, both diluted 1:100 in PBS-0.2% bovine serum albumin (BSA). For MUC2, the samples were then incubated with antirabbit secondary antibody conjugated with Alexa Fluor 568, diluted 1:300 in PBS-0.2% BSA.

Images were obtained with a Zeiss Apotome fluorescence microscope using 40X objective. The fluorescence intensity for ZO-1 was analyzed using the ImageJ software. The immunochemistry experiments were repeated three times, on different plates, incubated several weeks apart. Each time two wells were used for each condition, taking 8 images per well.

### 2.10 Statistical Analysis

The data were analyzed using R studio version 4.1.2. For all tests, the significance level was set at *p* < 0.05 (2 tailed) and all data were expressed as means of triplicates ± standard deviation (SD). The differences between the samples (protein hydrolysis, cell viability and permeability) were assessed by either two-way ANOVA followed by Tukey’s post hoc test or Kruskal–Wallis test followed by Dunn’s post hoc test as a nonparametric method (protein hydrolysis data).

## 3 Results

Two previously produced pea flours with contrasting levels of plant cell wall destructuration (R1, intact cell walls, and R2, ruptured cell walls) were used.^7^ Pea protein isolate (Prot) and extracted pea DF (Fib) were used as controls. To investigate the influence of dietary fiber on pea protein digestibility and on gut barrier health, these pea samples were subjected to *in vitro* gastrointestinal digestion, and the digesta obtained was applied to IPEC-J2 monolayers.

### 3.1 *In Vitro* Digestion and Characterization of the Digesta

**Figure 1:**
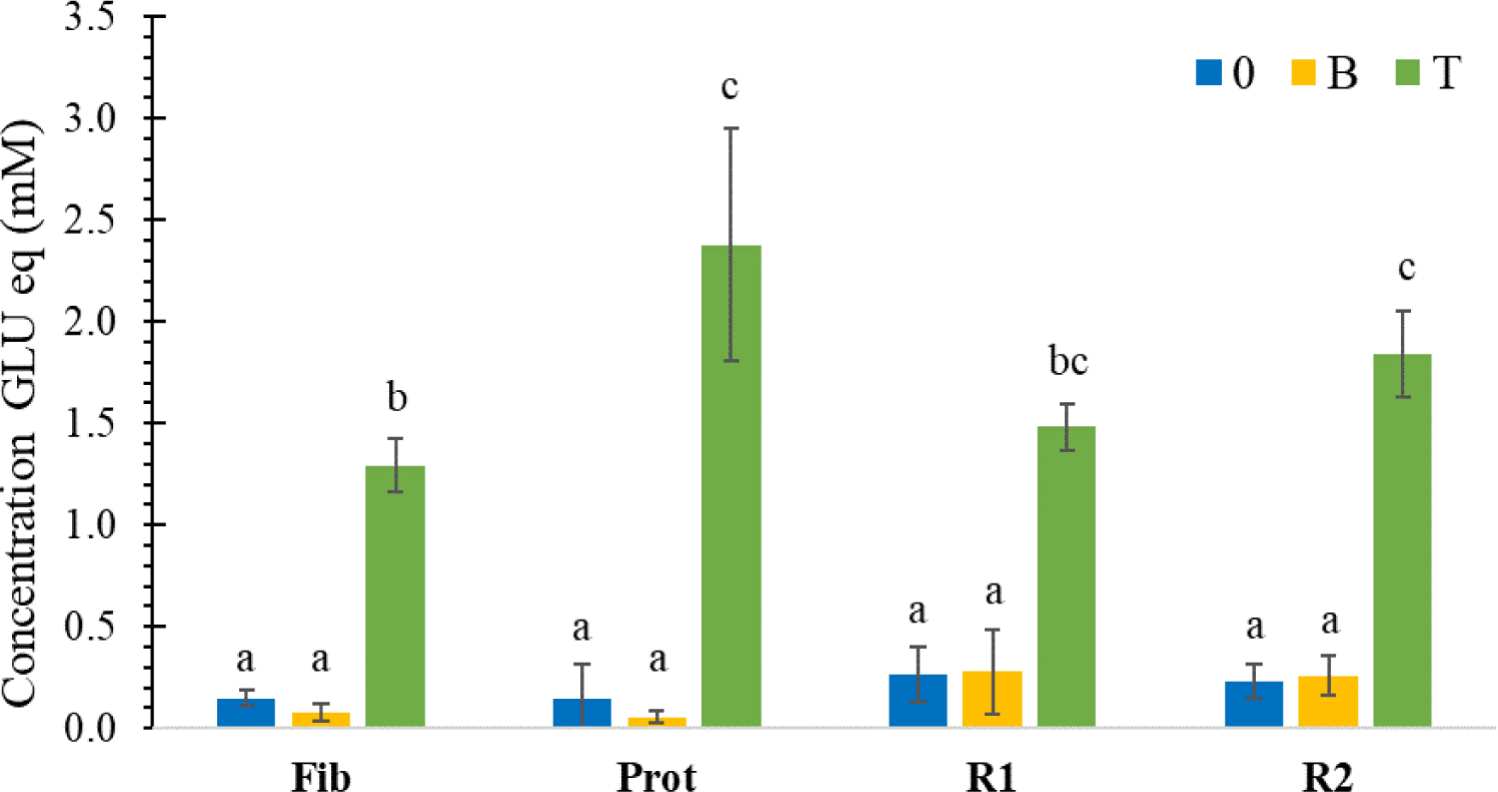
Protein digestibility expressed as glutamic acid equivalents (GLU eq in mM) corresponding to the free amino groups quantified by the OPA method for the raw materials with simulated digestive fluids (0), and gastrointestinal digestion of pea ingredients with bile only (B) or gastrointestinal digestion of pea ingredients with bile and enzymes (T). Fib, pea fibers; Prot, pea protein; R1, pea flour; and R2, reconstituted pea flour (containing Fib and Prot). Data are presented as mean and standard deviation (*n* = 3). The differences between the samples were analyzed using Kruskal–Wallis test followed by Dunn’s post hoc. Different letters indicate significant difference (*p* < 0.05).

Negligible amounts of free amino groups (amino acids or peptides) were detected in the raw pea materials (O) incubated in simulated digestion fluids only or during incubation in simulated fluids with bile but without enzymes (B) (Figure 1). Even though only 6.7 mg protein were present in the pea fiber samples (compared to 70 mg for the other pea ingredients),^7^ a significant amount of proteolysis products was detected; 1.35 mM glutamic acid equivalent for FibT, similar to 1.46 mM glutamic acid equivalent for R1T. Autohydrolysis of the digestive enzymes no doubt contributed to these high levels in FibT.^28,29^

The low level of proteolysis for R2 and the lack of proteolysis differences between R1 and R2 could be due to the detoxification process. Previously, we have reported a proteolysis of about 48.8 mM glutamic acid equivalent for R2 postgastrointestinal digestion.^7^ The heat treatment of all T samples followed by centrifugation may have caused protein precipitation and therefore removal of protein resistant to digestion and large peptides (see Figure S2).^30^ To evaluate this, the protein and peptide profile of the samples were visualized by SDS-PAGE.

**Figure 2:**
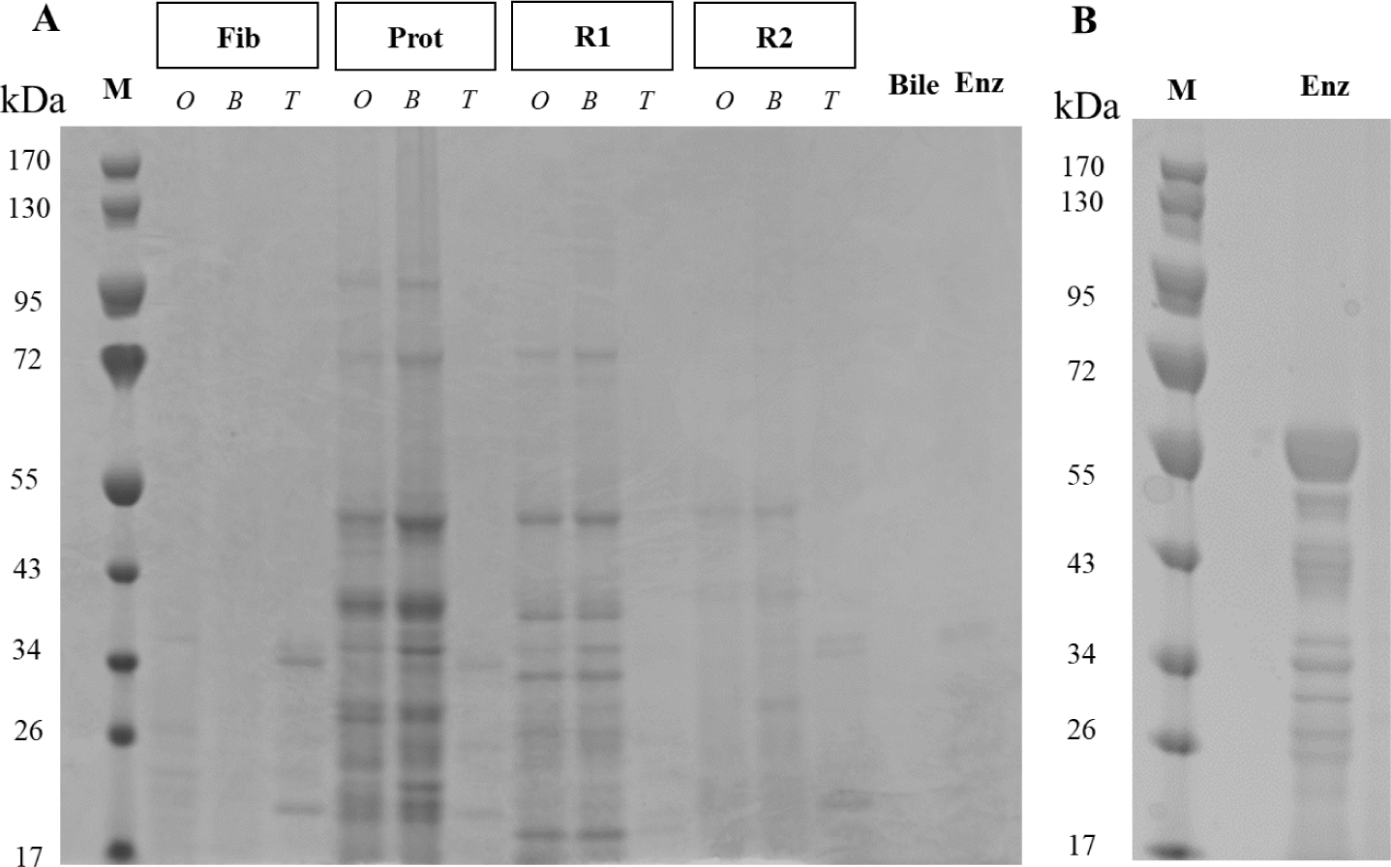
A) SDS-PAGE of the raw materials (*O*), incubated materials with bile only (*B*) and heat-treated, hydrolyzed digesta (*T*). Bile and enzymes (Enz), also heat treated, were used as controls. Fib, pea fibers; Prot, pea protein; R1, pea flour; and R2, reconstituted pea flour (containing Fib and Prot). B) Enzymes (Enz) without heat-treatment. M, marker.

Figure 2 shows that for most samples, little soluble protein remained after incubation with the exception of Prot (ProtO and B) and R1 (R1O and B). Bile salt appeared to slightly improve the solubilization of the proteins (bands more intense for ProtB, R1B and R2B) when comparing with O samples (bands more intense). Figure 2 clearly demonstrates few visible protein bands in heat-treated T samples or heat-treated enzyme sample. Proteins were therefore either hydrolyzed or discarded during the detoxification process (e.g., Enz in Figure 2A,B). However, some peptides, most likely produced by the autohydrolysis of the proteases (pepsin, as well as trypsin, chymotrypsin, and carboxypeptidase contained in the pancreatin), could be observed for Fib T. This confirms the results from the OPA assay in Figure 1. No protein was detected in the bile.

**Figure 3:**
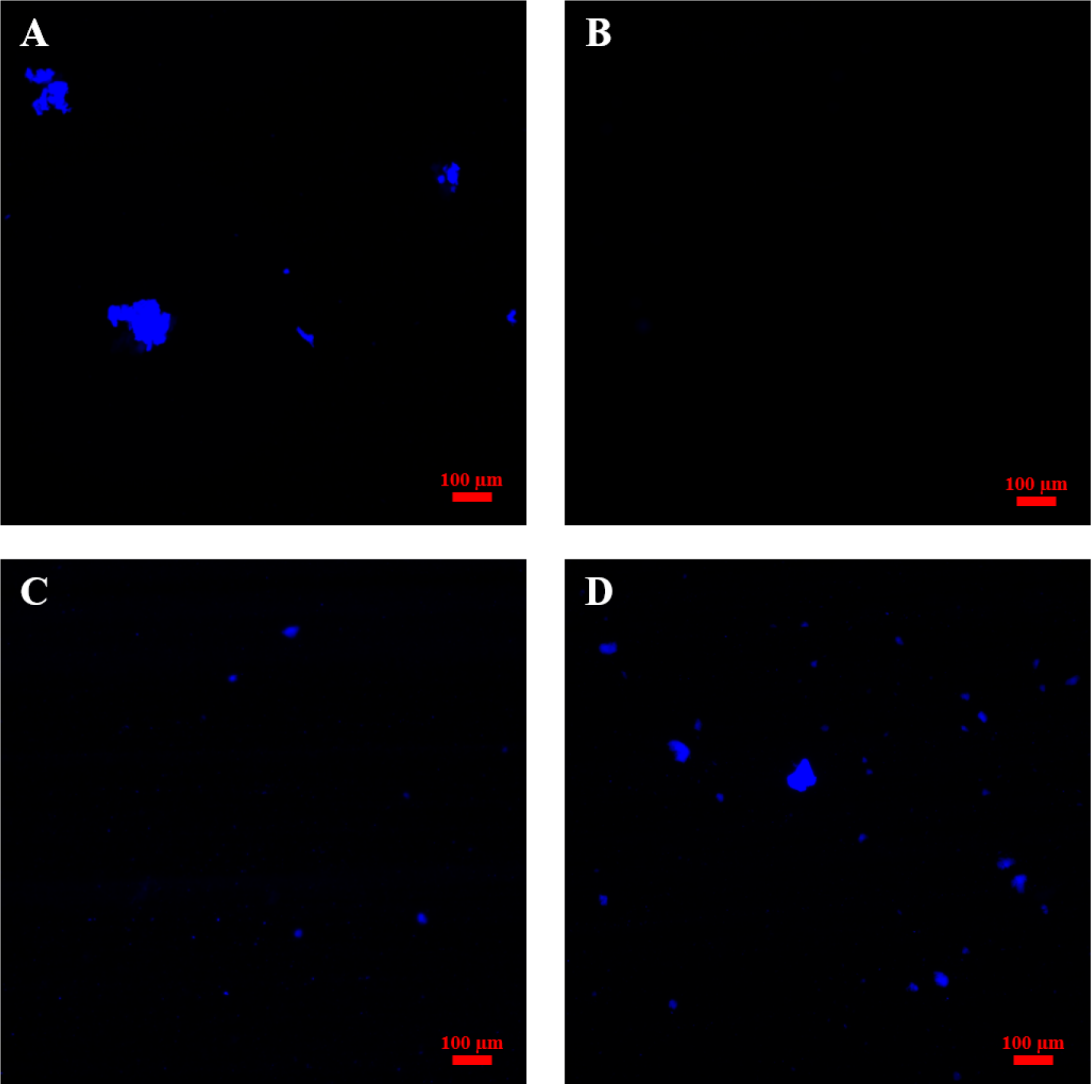
Microscopy images obtained with the Apotome following Calcofluor White staining for DF contained in the heat-treated digesta (T samples) of pea fibers (A), pea protein (B), R1 (C) and R2 (D). Scale bar = 100 μm.

In order to identify the DF and cell walls present in the aqueous phase of the detoxified digesta (heat-treatment and 1:10 dilution), the supernatant was stained with calcofluor white. As expected, no fiber was observed in the Prot sample (Figure 3B) and only a few solubilized cell wall components in the R1 sample (Figure 3C). On the other hand, larger cell wall fragments and broken cells could be visualized in the Fib and R2 samples (Figure 3A and D). Considering that R2 is made of Fib, this was expected. This approach revealed that despite centrifugation, heat-treatment and dilution, some cell wall fragments and even cells were still present in Fib, R1 and R2 samples and could potentially interact with the IPEC-J2.

### 3.2 Assessment of Cell Viability and Detoxification of the Digesta

Different detoxification conditions were tested, which revealed that a dilution of 1:10 with heat treatment was not cytotoxic to IPEC-J2 cells (Table 1). Indeed, values were close to the control (HBSS) with around 100% of viability, even after IPEC-J2 4 h of treatment with digesta. On the other hand, addition of a protease inhibitor (Pefabloc), with either a 1:5 or 1:10 digesta dilution, greatly compromised the cell viability, with between 15 and 36% of IPEC-J2 remaining viable after 1 h of incubation with the digesta (see Figure S1). Heat treatment also reduced the toxicity from bile (possibly by degrading and/or precipitating the bile salts) and not surprisingly reduced the toxicity of digestive enzymes.

**Table 1:** IPEC-J2 Viability after 1, 2, or 4h Treatment with Pea Ingredients, Gastrointestinal Pea Digests and Digestion Controls^a^

Cell viability(% vs. Control)									
Samples Dilution (v/v)		1h			2h			4h	
Control(cells in HBSS)		100.0 ± 9.1			100.0 ± 9.1			100.0 ± 6.5	
Caontrol bile									
1:5		99.1 ± 7.5			111.2 ± 1.8			101.5 ± 8.3	
1:10		96.3 ± 4.1			107.0 ± 12.9			98.7 ± 13.8	
Control enzymes									
1:5		118.5 ± 3.7			119.6 ± 7.3			103.9 ± 12.1	
1:10		107.4 ± 2.9			115.8 ± 16.9			102.6 ± 8.2	

		1h			2h			4h	
		*B*	*T*		*B*	*T*		*B*	*T*
Fib									
1:5	95.6 ± 4.7	111.0 ± 6.0	119.6 ± 6.8	120.7 ± 1.7	120.7 ± 1.7	121.3 ± 5.0	90.9 ± 14.2	103.3 ± 17.2	99.1 ± 17.7
1:10	95.0 ± 13.6	112.7 ± 12.0	114.9 ± 10.7	105.5 ± 2.7	125.3 ± 9.8	114.8 ± 13.2	93.6 ± 5.6	108.5 ± 13.9	109.9 ± 13.9
Prot									
1:5	102.1 ±4.1	106.5 ± 5.4	106.9 ± 12.3	104.7 ± 4.2	113.7 ± 1.7	118.1 ± 2.6	97.9 ± 19.9	100.0 ± 19.3	96.8 ± 19.8
1:10	99.3 ± 8.2	106.7 ± 3.8	112.5 ± 6.4	110.4 ± 1.8	126.1 ± 5.0	123.7 ± 12.8	93.5 ± 13.7	102.7 ± 9.0	104.4 ± 12.1
R1									
1:5	95.2 ± 3.4	111.0 ± 15.3	125.4 ± 12.3	108.8 ± 3.6	119.7 ± 8.1	124.3 ± 1.3	98.5 ± 4.4	112.5 ± 5.7	113.6 ± 7.5
1:10	104.2 ± 5.1	114.4 ± 5.2	122.4 ± 8.5	105.3 ± 4.1	122.2 ± 9.4	124.2 ± 13.2	97.8 ± 4.6	106.7 ± 9.6	113.8 ± 7.4
R2									
1:5	99.1 ± 6.9	108.0 ± 11.7	116.1 ± 10.6	109.6 ± 0.4	127.9 ± 1.7	128.5 ± 0.5	100.5 ± 10.6	112.3 ± 4.4	115.9 ± 9.8
1:10	102.4 ± 4.4	142.2 ± 8.8	122.4 ± 14.6	106.4 ± 6.0	133.0 ± 8.4	133.1 ± 20.0	97.0 ± 6.8	111.1 ± 15.5	112.2 ± 10.5

a Fib, pea fibers; Prot, pea protein; R1, pea flour; and R2, reconstituted pea flour (containing Fib and Prot). O, samples incubated (for 1, 2 or 4h) in simulated fluids without bile or enzymes; B, samples incubated with simulated fluids and bile but without enzymes; T, samples incubated with both bile and enzymes (heat-treated). Values are means of triplicate, ± standard deviation. There was no statistical difference between the samples as analyzed by two-way ANOVA and the Tukey’s post-hoc test.

### 3.3 Evaluation of IPEC-J2 Permeability

**Figure 4:**
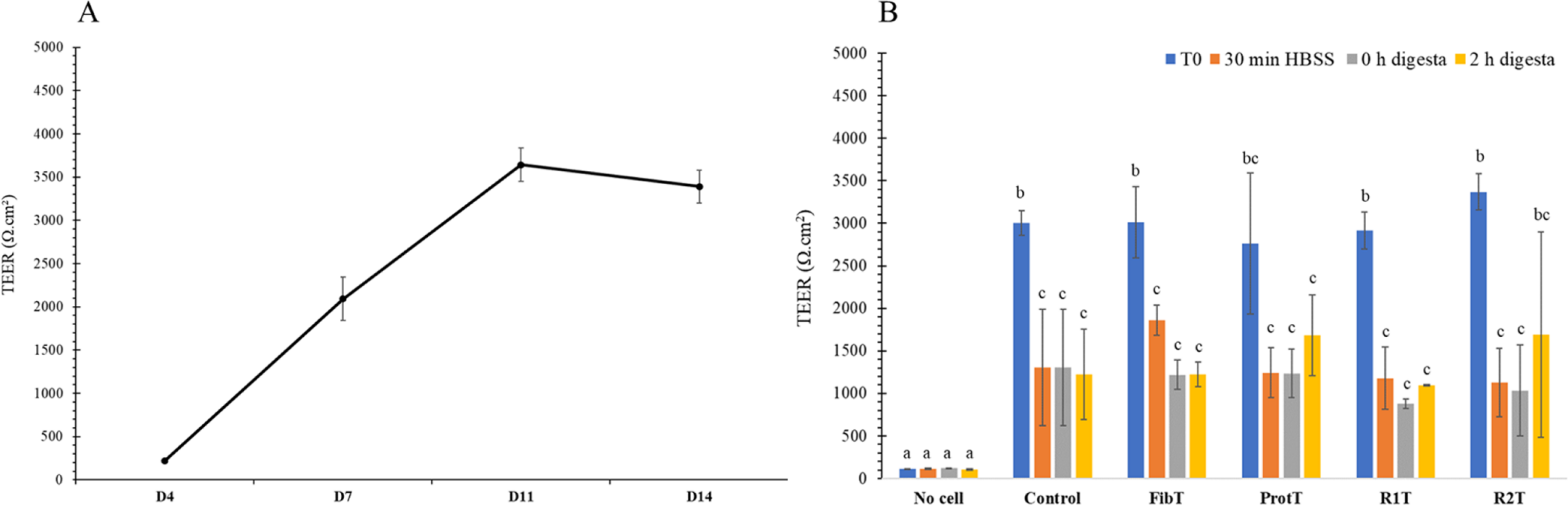
Time course of TEER measurements of IPEC-J2 (A) during their growth and differentiation and (B) after incubation with HBSS (Control) or the different heat-treated digesta (FibT for pea fibers, ProtT for pea protein, R1T for R1, and R2T for R2). Data are presented as mean and standard deviation (*n* = 3). The effect of the treatment (HBSS or digesta) and time were analyzed using two-way ANOVA followed by Tukey’s posthoc test. Different letters indicate significant difference (*p* < 0.05).

The TEER values measured during the growth and differentiation of the epithelial cells used in this work are presented in Figure 4. As expected, the TEER increased overtime until stabilization at day 14, demonstrating the barrier integrity of the IPEC-J2 monolayers. At day 14, the various digesta were applied to monolayers washed with HBSS. TEER measurements were consistent among the different wells, with an average value of 3100 ± 684 Ω.cm^2^ after 14 days of culture. The change of media for HBSS created a decrease in TEER that persisted overtime (a reduction to 1342 ± 297 Ω.cm^2^). Some TEER increases were observed after 2 h of incubation for some samples (ProtT and R2T), but overall no significant differences were noticed compared to the control.

**Figure 5:**
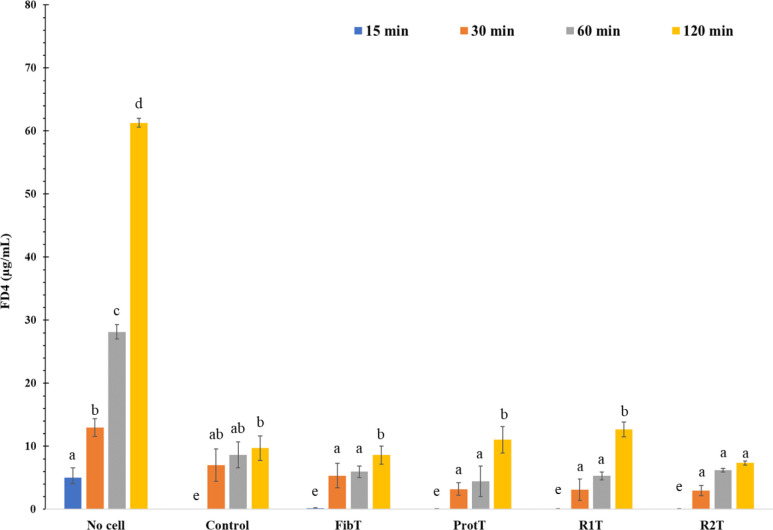
Overtime passage of FD4 (in ng/μL) across the IPEC-J2 monolayers after incubation with no cell, medium only (control) or digesta from pea fibers (FibT), pea protein (ProtT), R1T, and R2T.

Figure 5 indicated a limited passage of FD4 across the IPEC-J2 monolayers when monolayers were in contact with the media only (C = control), with a slight increase overtime. The FD4 concentration in the basal compartment for the digesta was similar to the FD4 concentration found for the control, only R2T showed a significant difference compared to the other sample at 120 min. The *P*_app_ of the FD4 was on average of 2.5 × 10^–7^ cm.s^–1^ for both the monolayers control and the monolayers treated with pea digesta at the end of the incubation period.

**Figure 6:**
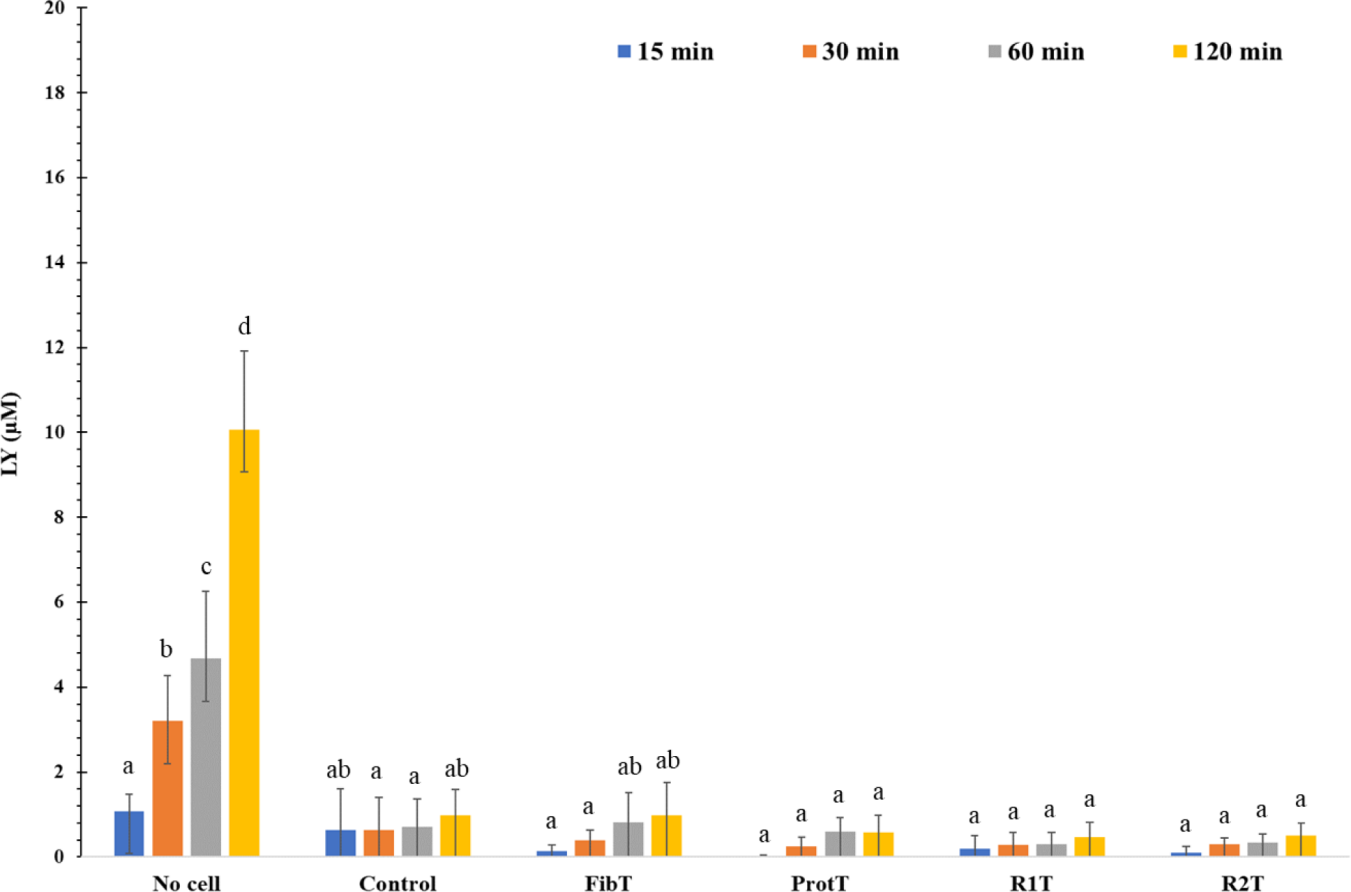
Passage of LY (in μM) over 120 min, at day 14 of culture, across the IPEC-J2 monolayers after incubation with no cell, medium only (control) or digesta from pea fiber (FibT), pea protein (ProtT), R1 (pea flour) and R2 (reconstructed pea flour). Data are presented as mean and standard deviation (*n* = 3). The effect of the treatment (HBSS or digesta) and time were analyzed using two-way ANOVA followed by Tukey’s posthoc test. Different letters indicate significant difference (*p* < 0.05).

The diffusion of LY through the monolayers was not statistically different for monolayers treated with digesta samples (Figure 6). The *P*_app_ of the LY was low with an average value after 2 h of incubation of 3.2 × 10^–6^ cm.s^–1^ for the control and 1.1 × 10^–6^ cm.s^–1^ for the pea digesta condition. This indicates that little paracellular transport of this hydrophilic molecule took place and that the epithelial barrier was preserved after exposure to the digesta.

**Figure 7:**
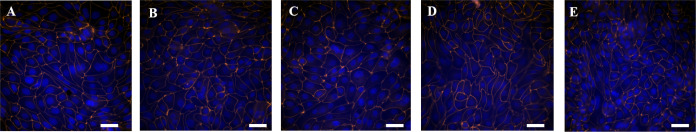
Representative images of ZO-1 immunostaining of IPEC-J2 after 2 h incubation with cell media (A) or digested pea fibers (B), pea protein (C), R1 (D), and R2 (E). ZO-1 immunostaining in red and DAPI counterstaining of nuclei in blue. Scale bar = 50 μm.

To observe the microstructure of the IPEC-J2 monolayers postdigesta treatment, immunohistochemistry for the tight junction proteins ZO-1 was performed (Figure 7). The images confirm the permeability assays; the presence of DF did not affect the integrity of the IPEC-J2. The IPEC-J2 monolayers appeared compact and neighboring cells adherent to each other, with ZO-1 staining visible around the cells, regardless of pea digesta.

## 4 Discussion

The study revealed that DF from pea, regardless of their structure (mainly intact vs. ruptured cell walls), did not compromise the barrier function of jejunal epithelial cells when using IPEC-J2 as a model.

### 4.1 Effect of Digested Pea Ingredients on Gut Barrier Function

*In vitro* intestinal epithelial models are good tools to shed light on interactions of food compounds with the gut barrier function. Contrary to the commonly used colon adenocarcinoma cell line-2 (Caco-2), the jejunal IPEC-J2 are not cancerogenic^20,21,33^ which suggests that they more closely simulate the physiology and the metabolism of the jejunal where absorption of the proteolysis products occurs.^34^ Despite using improved conditions of culture, notably by substituting fetal bovine serum by porcine serum, the TEER values recorded at day 14 were high (on average around 3391 ± 188 Ω/cm^2^).^21^ These values are much higher than physiological ones (50 Ω/cm^2^) but of the same order of magnitude to the TEER values found in the literature for IPEC-J2.^20,35,36^

Diffusion of labeled molecules can also be used to assess the integrity of the intestinal barrier in the presence of DF and other compounds (molecules or structures such as cell wall fragments), for instance monitor the transport of 4000 Da FD4 and 521.8 Da LY from the apical to the basolateral compartments of polarized intestinal cells.^31,32,38^ These assays give an appraisal of the paracellular flow, where hydrophilic molecules passively diffuse between cells based on their size.^37^ Therefore, in our study, the jejunal barrier was not altered when exposed to DF, with molecules of either 522 or 4 000 Da unable to cross between the cells. The values of *P*_app_ for the FD4 (2.5 × 10^–7^ cm.s^–1^) were in the range of the one found in the literature for the Caco-2, with *P*_app_ between 0.27 and 19.9 × 10^–7^ cm.s^–1^.^31^ Similarly, the reported *P*_app_ of LY in Caco-2 ranged between 0.16 to 3.0 × 10^–6^ cm.s^–1^, whereas we observed a *P*_app_ of 3.3 × 10^–6^ cm.s^–1^ for the control and 1.1 × 10^–6^ cm.s^–1^ for the pea digesta. Experiments performed on Ussing Chamber lead to slightly higher values with average *P*_app_ for human small intestine of 4.0 × 10^–6^ cm.s^–1^.^32^ The TEER measurements are consistent with our FD4 and LY diffusion data, and the values did not decrease compared to the control even after 2 h of incubation with the pea digesta.

In our study, a trend, although not statically significant, was observed, suggesting a potential positive effect of DF (or at least no detrimental impact) after 2 h of incubation. Similarly, no difference was detected with the control, with regard to a specific protein involved in the cell adhesion complexes regulating the diffusion of molecules between intestinal cells (ZO-1). ZO-1 interacts with a number of tight junction-associated proteins, such as occludin, which together regulate the passage of molecules via the paracellular pathway.^41^ ZO-1 as a scaffolding protein is particularly critical in maintaining the gut barrier. *In vivo* piglets fed pea fiber had higher villous height and ZO-1 mRNA levels compared to the piglets fed the control diet (with no added DF) demonstrating an improved intestinal barrier function.^18^ These results suggest that DF may have stimulated ZO-1 production. However, in our acute study, we were unable to detect any significant increase in ZO-1 intensity (see Figure S3), possibly because changes to ZO-1 may require dietary intervention over several days/week rather than 2 h.

### 4.2 Validation of Our Approach to Study the Impact of DF on Gut Barrier Function

Preparation of the digesta for use in cell culture studies is a critical point that needs careful consideration depending on the research question and objective and may require a combination of detoxification strategies (e.g., dilution and heat-treatment as used in this study).^23^ Different compounds present in the digesta (bile salts, enzymes and other proteins, and DF) may affect IPEC-J2 viability as well as the integrity and the permeability of the monolayer.^23^ Even though proteins were removed from the digesta via the detoxification process (Figures 2 and S2), our samples contained small particles and molecules, generated from the hydrolysis process of pea ingredients (peptides, cell wall fragments and solubilized polysaccharides) that may have influenced the permeability of the cell monolayer (see Figures 2 and 3). It is well-known that certain elements present in digesta can be toxic for intestinal epithelial cells.^23^ As shown in Figure 2, the digesta contained not only DF but also products of the protein hydrolysis, i.e., peptides. Some peptides maybe bioactive and have a positive impact on IPEC-J2. However, even though DF (Fib, R1 or R2 digested samples) were present in the samples the permeability and integrity of the IPEC-J2 were preserved.

**Figure 8:**
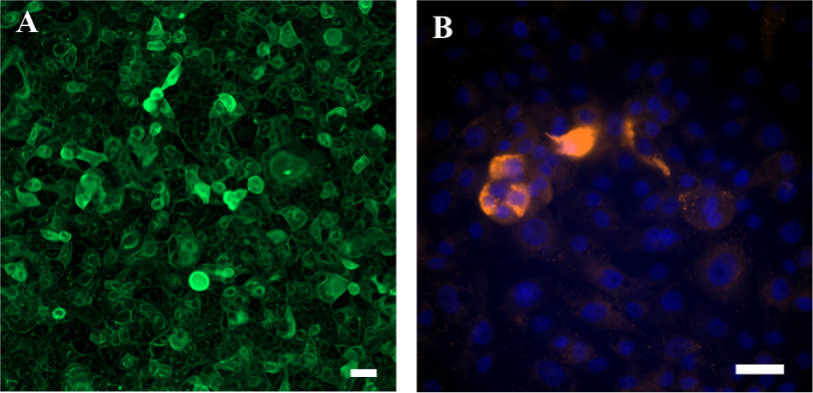
IPEC-J2 labeled with either wheat germ agglutinin in green (A) or MUC2 antibodies in red (B). Note the cells nuclei stained in blue with DAPI in image B. Scale bar = 50 μm.

A possible explanation for this could be that the mucus layer protected the cells from the compounds contained in the digesta.^20,33,42,43^ Several studies failed to identify mucus-secreting cells within the IPEC-J2.^20,44^ It remains therefore unclear if the IPEC-J2 cells actually produce mucin, and in sufficient quantity to cover the entire monolayer. The culture conditions (e.g., serum origin, passage, seeding density, and insert with or without collagen) certainly play a critical role in the mucin production and the complexity of the mucus layer formed. In our lab, we succeeded in visualizing some glycoproteins, and possibly mucin, produced by the IPEC-J2 using the culture conditions described above (Figure 8). These images show glycoproteins stained with wheat germ agglutinin (unspecific staining of glycoproteins, Figure 8A) and MUC2 specifically (Figure 8B), although MUC2 was observed only in a few areas on the cell monolayers. The literature gives inconsistent data where mucin is not always detected.^20,45−48^ Other authors suggested that IPEC-J2 possess a glycocalyx but no mucin.^49^ In any case, this glycocalyx could have prevented digested pea components, particularly DF, damaging the IPEC-J2 cells.

### 4.3 Hypothesis on Nutrients Absorption and Perspectives

DF, by affecting the properties of mucus or altering epithelial physiology, could hinder or promote the absorption of nutrients.^14,18^ Studies performed in pigs and humans showed that DF can influence gut health and functionality but this effect relies on their physicochemical properties rather than their composition.^6,39^ Moreover, the impact of DF would depend on many other factors such as the site of the gastrointestinal tract considered (e.g., mucus secretion, abrasive effect, or fermentation by microorganism), the development stage (unweaned baby or young animal, adult vs elderly) and health status (e.g., inflammation or bowel disease) of the host.^6,40^ Therefore, DF could play a key role in promoting optimal gut health, and efficient digestibility particularly in the small intestine, the primary site of nutrients hydrolysis and absorption. From these results, it would be expected that the absorption of proteolysis products would take place in the presence of pea DF assuming that the proteins is bioaccessible (proteins available for hydrolysis and the products available for absorption).^50^ However, it is unsure how long the digesta would be in contact with the cells in the jejunum, the transit time will depend on the digesta characteristics (water, lipid, protein, and DF contents as well as particle size and the overall viscosity).^7,51^ Moreover, epithelial cells and their environment may adapt and as a result, modulate nutrient absorption. For instance, more mucus may be secreted and cells renewed in response to the presence of DF. Finally, even though less important than in the colon, a microbiota exists in the jejunum and it may feed on these DF, thereby changing the epithelial cells microenvironment.^52^ It is likely that DF have different effect on the gut barrier based on the location within the gastrointestinal tract (jejunum, ileum or colon); each section having a specific morphology (mucus layer) and microbiota community.^53,54^ All of the processes described here are dynamic and interconnected, making it challenging to study in a 2D static *in vitro* model. The main limitation of the IPEC-J2 is their lack of full interactions with neighboring cells and tissues which could be partially achieved with a three-dimensional cell culture such as organoids or organ-on-chip.^55,56^ Another model, that could complement these ones, is Ussing Chamber.^32^ The latter has the advantage to simulate closely the *in vivo* conditions (e.g.; mucus layer, cells types); however, it requires sacrifice of animals while increasing the variability between measurements (less controlled conditions than with the IPEC-J2).

The combination of *in vitro* digestion and IPEC-J2 used in this study provided further insights on the degradation of pea ingredients during digestion and how the solubilized DF interacted with jejunal cells. Monitoring the uptake of the products of proteolysis is challenging with cell culture, notably because of the amino acids and peptides present in media and generated by the cells themselves. Therefore, using a range of methods that describes the integrity and permeability of the cell monolayer is a valuable tool.

Additional studies are required to investigate in more detail the structure and supramolecular assembly of the polysaccharides solubilized in the digesta (R1 and R2). Also, a thorough characterization of the potential mucus layer covering the IPEC-J2 is necessary. How close are its composition and thickness compared to the jejunum? How does DF modulate it and the subsequent cell physiology and functionality?

Finally, investigating the fate of the digesta in the colonic compartment and their effect on the microbiota would complement this work. It would provide a comprehensive view of the events occurring along the gastrointestinal tract when consuming pea fibers under different forms and help understanding the health benefit of consuming DF from legumes such as pea.

## Supporting Information Available

Figure S1: Percentage of cell viability of the IPEC-J2 after exposure with digesta containing Pefabloc (1 mM). Figure S2: SDS-PAGE of the digested pea materials obtained from our previous study clearly showing the proteins present in the sample after incubation without (B) or without (T) enzymes. Figure S3: Light intensity measured for ZO-1 as a function of the number of nuclei (PDF)
