# Supplementary material for: In Vitro Evaluation of Intestinal Barrier Function after Exposure to Digested Pea Ingredients—Food Matrix Effect
Source: J Agric Food Chem. 2024 Dec 16;73(1):584–94. doi: 10.1021/acs.jafc.4c09963 (PMC11726683; doi:10.1021/acs.jafc.4c09963)
Supplement: Supplementary file 1 — jf4c09963_si_001.pdf [file jf4c09963_si_001.pdf]

Supporting Information

In vitro evaluation of intestinal barrier function after exposure to digested pea ingredients - food matrix effect

Marie-Hélène Perruchot[1], Gaëlle Boudry[2], Frédérique Mayeur-Nickel[1], Maurane Grondin[1], Sandra Wiart-Letort[1], Linda Giblin[3], Myriam M.-L. Grundy[1]*

[1]PEGASE, INRAE, Institut Agro, 35590 Saint Gilles, France.

[2]NUMECAN, INSERM, INRAE, Université de Rennes, 35590 Saint Gilles, France.

[3]Teagasc Food Research Centre, Moorepark, Fermoy, Co Cork, P61 C996, Ireland

*Email: myriam.grundy@inrae.fr

Figure S1: Percentage of cell viability of the IPEC-J2 after exposure with digesta containing Pefabloc® (1 mM).

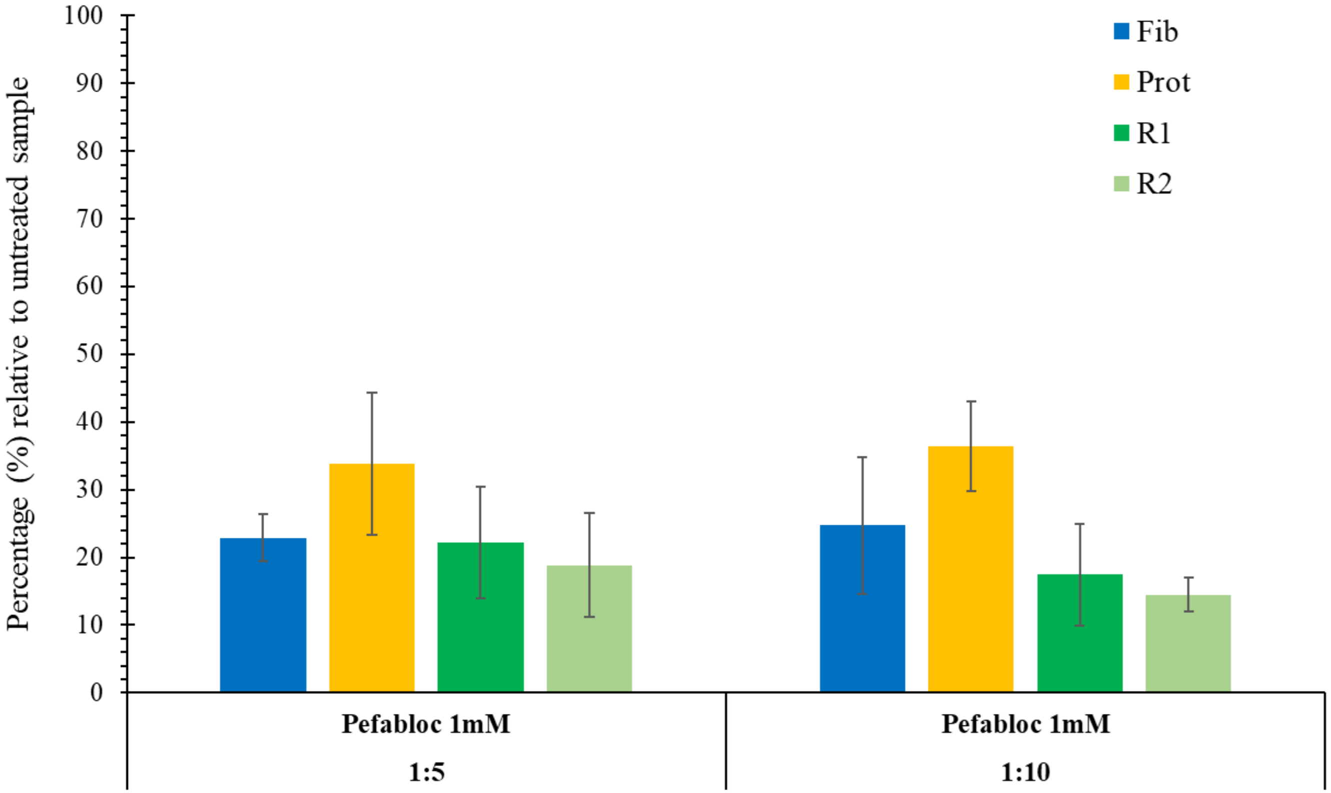

Fib, pea fibres; Prot, pea protein; R1, pea flour; and R2, reconstituted pea flour

Figure S2: SDS-PAGE of the digested pea materials obtained from our previous study[1] clearly showing the proteins present in the sample after incubation without (B) or without (T) enzymes.

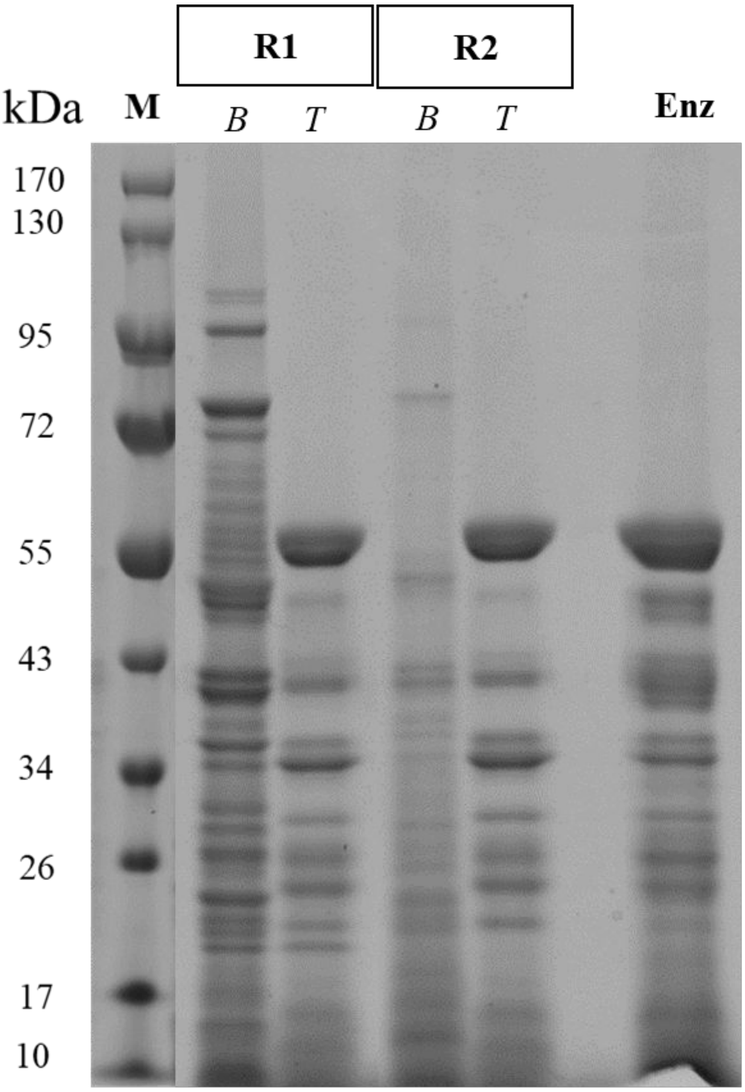

Figure S3: Light intensity measured for ZO-1 as a function of the number of nuclei.

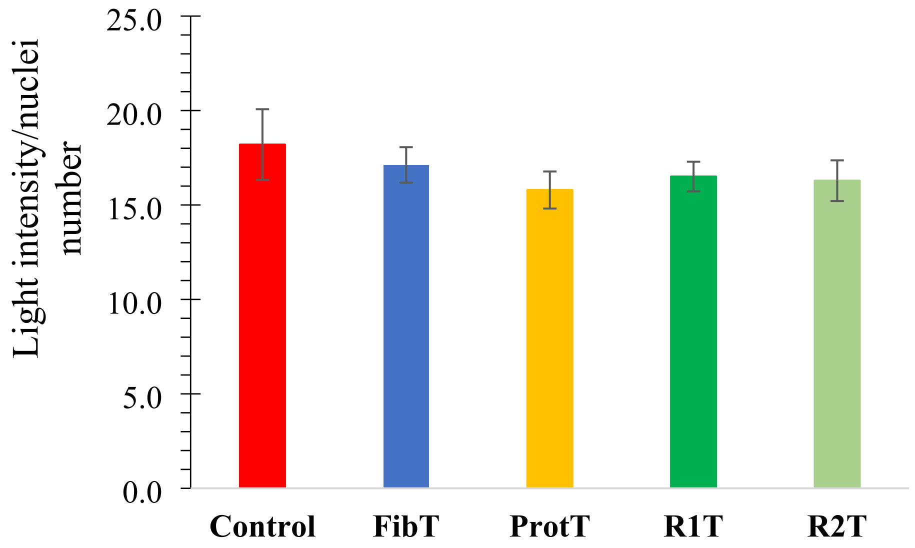

References

1. Grundy, M. M.-L.; Labarre, J.; Mayeur-Nickel, F.; van Milgen, J.; Renaudeau, D., An *in vitro* and *in vivo* approach to characterise digesta from pigs fed different forms of pea flour. *J Anim Sci* **2023**, *101*, 1-11.
